# Supplementary figures and images for: Members of the methanotrophic genus Methylomarinum inhabit inland mud pots
Source: PeerJ. 2016 Jul 12;4:e2116. doi: 10.7717/peerj.2116 (PMC4950536; doi:10.7717/peerj.2116)

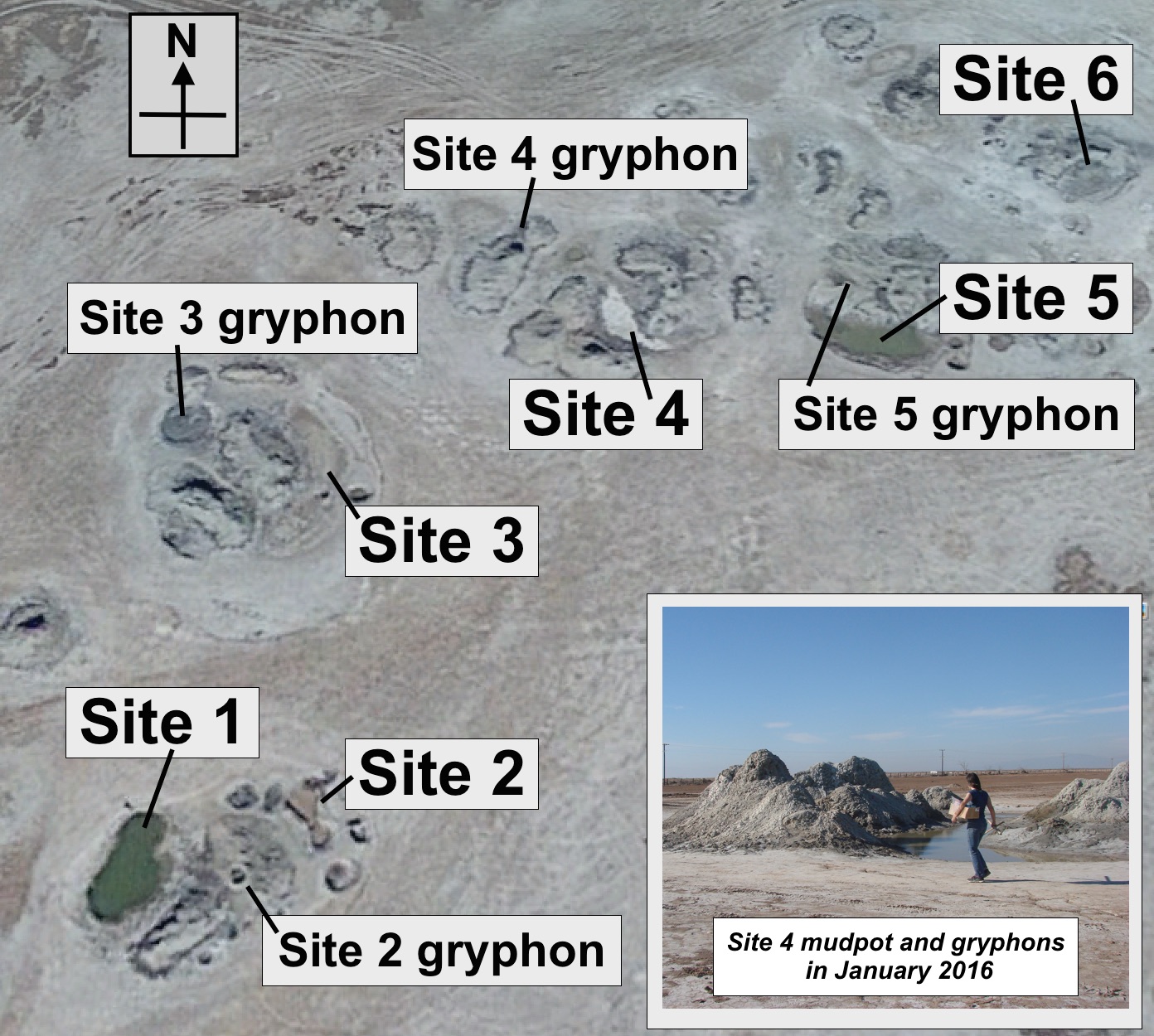

Supplement: Supplemental Information 1 — In 2012, samples were collected from the light brown pot at site 2 and the dark grey pot at site 3. In 2016, samples were collected from water, sediment, and gryphons associated with Sites 1–6, as indicated in Table 1. Inset: Pots were roughly 2–4 meters in diameter; gryphons were roughly 2–2.5 m tall. [file peerj-04-2116-s001.jpg]

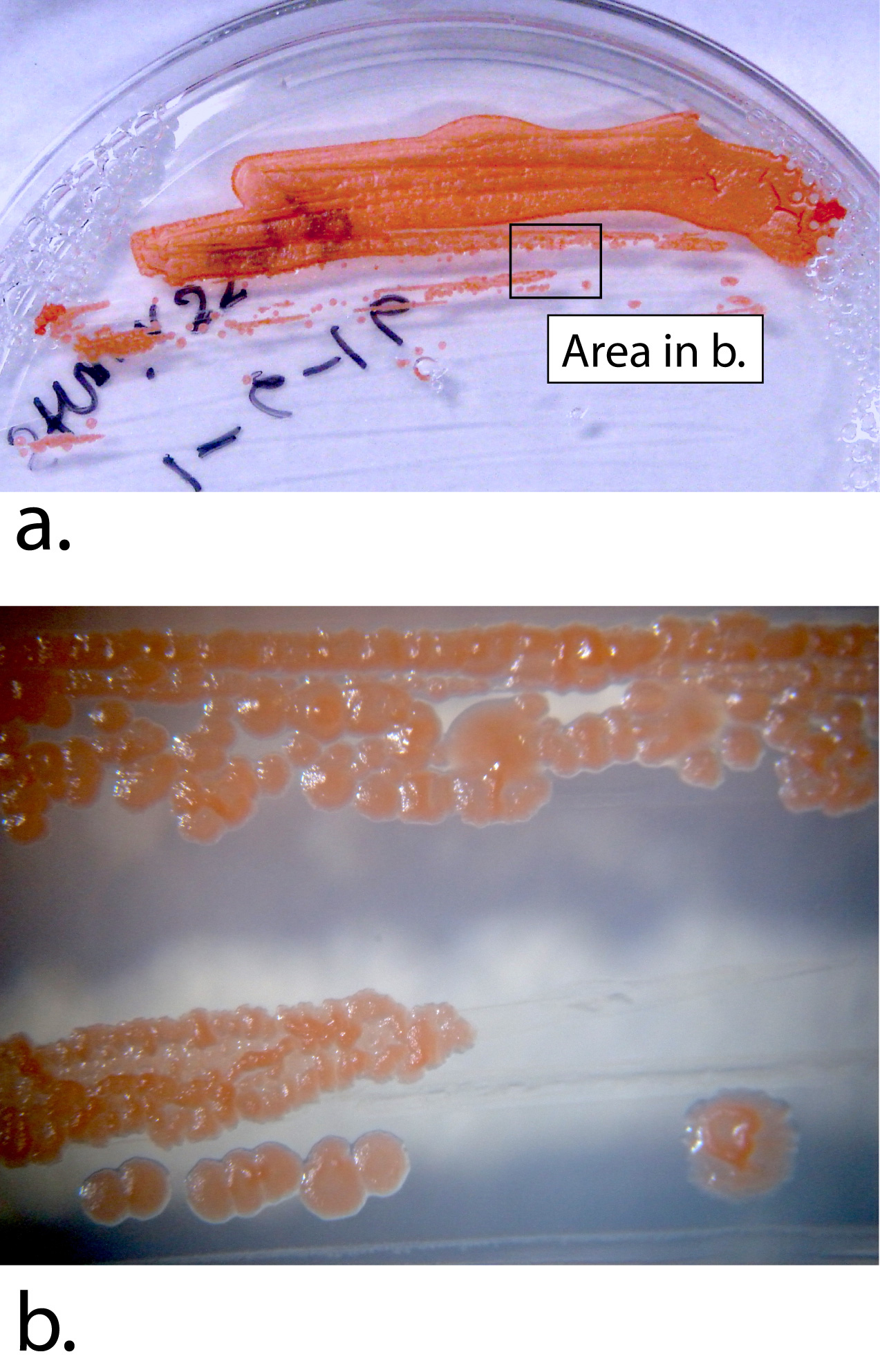

Supplement: Supplemental Information 2 — Colonies required approximately 2 weeks to become visible on solid modified NMS plates incubated at 22 °C. Between 2 and 4 weeks growth, colonies became rough in appearance and non-uniform pink pigmentation developed. (A) View of colonies by eye. (B) View of colonies by stereoscope. [file peerj-04-2116-s002.jpg]

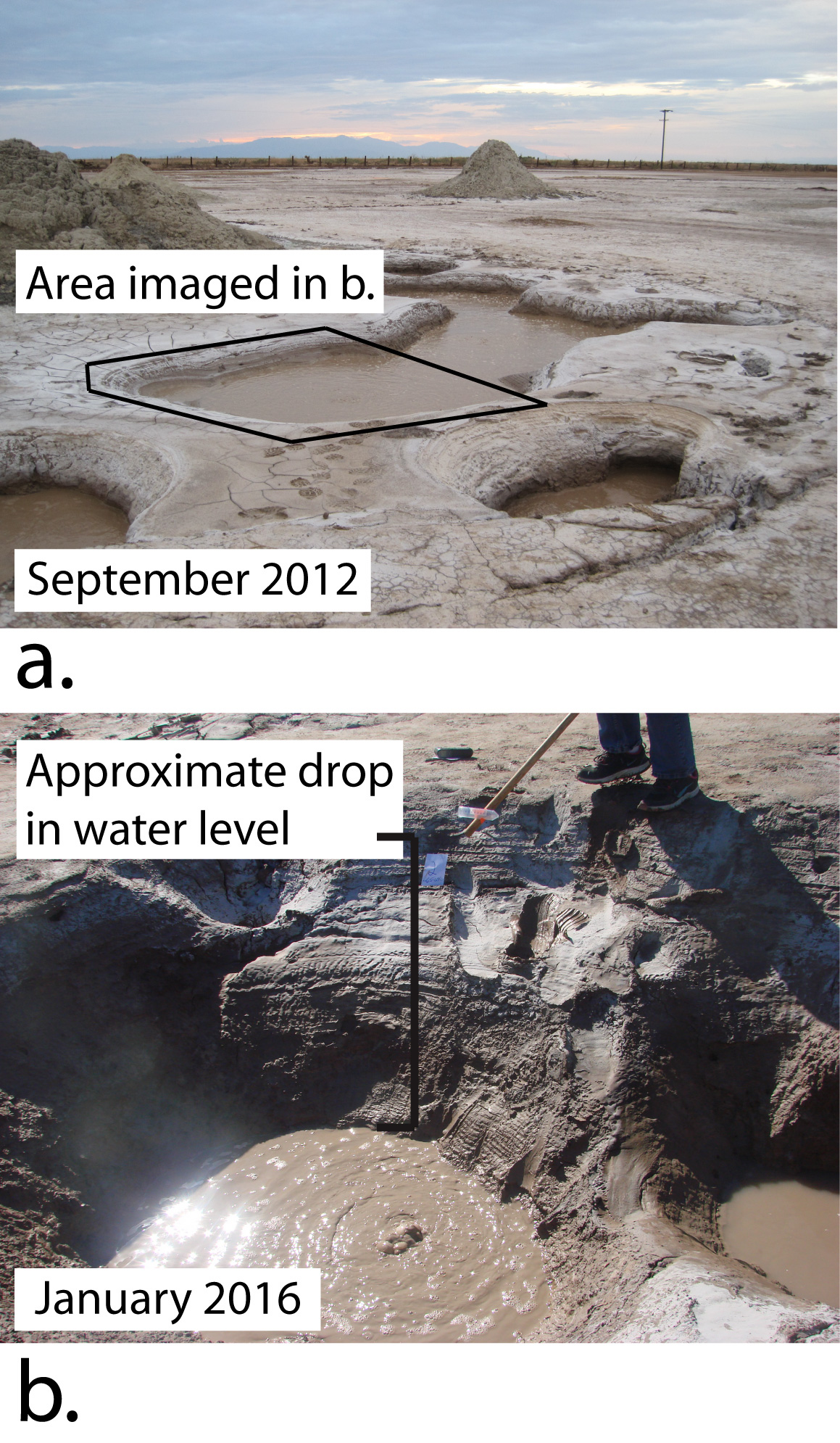

Supplement: Supplemental Information 3 — (A) In 2012, water levels at Site 2 were within 0.2 m of ground level. (B) Water levels were significantly lower in 2016, approximately a meter below ground level. [file peerj-04-2116-s003.jpg]
